# Anthelminthic and antimicrobial effects of hedge woundwort (*Stachys sylvatica* L.) growing in Southern Kazakhstan

**DOI:** 10.3389/fphar.2024.1386509

**Published:** 2024-05-06

**Authors:** Aigerim Z. Mukhamedsadykova, Martyna Kasela, Kaldanay K. Kozhanova, Zuriyadda B. Sakipova, Wirginia Kukuła-Koch, Aleksandra Józefczyk, Łukasz Świątek, Barbara Rajtar, Magdalena Iwan, Przemysław Kołodziej, Agnieszka Ludwiczuk, Gulnara M. Kadyrbayeva, Gulnur N. Kuntubek, Aliya S. Mamatova, Anna Bogucka-Kocka, Anna Malm

**Affiliations:** ^1^ Department of Engineering Disciplines of Good Practices, School of Pharmacy, Kazakh National Medical University, Almaty, Kazakhstan; ^2^ Department of Pharmaceutical Microbiology, Medical University of Lublin, Lublin, Poland; ^3^ Department of Pharmacognosy, Medical University of Lublin, Lublin, Poland; ^4^ Department of Pharmacognosy with Medicinal Plant Laboratory, Medical University of Lublin, Lublin, Poland; ^5^ Department of Virology with Viral Diagnostics Laboratory, Medical University of Lublin, Lublin, Poland; ^6^ Department of Toxicology, Medical University of Lublin, Lublin, Poland; ^7^ Department of Biology and Genetics, Medical University of Lublin, Lublin, Poland; ^8^ Department of Microbiology and Virology, Kazakh National Medical University, Almaty, Kazakhstan

**Keywords:** *Stachys sylvatica L.*, ultrasonic-assisted hydroethanolic extraction, chlorogenic acid, luteolin derivatives, verbascoside, bioactivity, anthelminthic activity

## Abstract

The *Stachys* L. genus has been widely used in traditional medicine in many countries throughout the world. The study aimed to investigate the chemical composition and bioactivity of the hydroethanolic extract (50% v/v) obtained by ultrasonication from the aerial flowering parts of *Stachys sylvatica* L. (SSE) collected in Almaty region (Southern Kazakhstan). According to RP-HPLC/PDA analysis the leading metabolites of the SSE belonged to polyphenols: chlorogenic acid and its isomers (2.34 mg/g dry extract) and luteolin derivatives (1.49 mg/g dry extract), while HPLC-ESI-QTOF-MS/MS-based qualitative fingerprinting revealed the presence of 17 metabolites, mainly chlorogenic acid and its isomers, flavonoid glycosides, and verbascoside with its derivatives. GC-MS analysis of the volatile metabolites showed mainly the presence of diterpenoids and fatty acid esters. A reduction in the viability of nematodes *Rhabditis* sp. was obtained for the SSE concentration of 3.3 mg/mL, while 11.1 mg/mL showed activity comparable to albendazole. The SSE exhibited higher activity against Gram-positive (MIC = 0.5–2 mg/mL) than Gram-negative bacteria and yeast (MIC = 8 mg/mL), exerting bactericidal and fungicidal effects but with no sporicidal activity. The SSE showed some antiviral activity against HCoV-229E replicating in MRC-5 and good protection against the cytopathic effect induced by HHV-1 in VERO. The SSE was moderately cytotoxic towards human cervical adenocarcinoma (H1HeLa) cells (CC_50_ of 0.127 mg/mL after 72 h). This study provides novel information on the SSE extract composition and its biological activity, especially in the context of the SSE as a promising candidate for further antiparasitic studies.

## 1 Introduction

*Stachys sylvatica* L., commonly known as hedge woundwort, is a perennial plant that belongs to the Lamiaceae family consisting of approximately 370 species and about 435 taxa (Güner et al., 2021). It is characterized by an erect, hirsute, tall stem that can grow up to 100 cm. It has opposite, cordate leaves, which are sparsely hirsute. It blooms from June to August, producing reddish-purple flowers organized in verticillasters. The leaves are responsible for the plant’s unpleasant smell (Alizadeh et al., 2020). Plants from the *Stachys* L. genus can be found in both hemispheres, including the Mediterranean Region, southwest Asia as well as in South and North America and North Africa, except for New Zeland and Australia (Tunçtürk et al., 2019; Güner et al., 2021; Kanjevac et al., 2023). *S. sylvatica* L. growth area in Kazakhstan includes Altai, Tarbagatai, the Dzungarian Alatau and the ridges of the Northern Tian Shan: Zailiysky, Kungei, Terskei Alatau and Ketmen. *S. sylvatica* L. prefers wet and shady forest areas, the banks of rivers and streams, often places situated at high altitudes (Hajdari et al., 2012; Alizadeh et al., 2020; Apostolescu et al., 2023).

It is worth notifying that the *Stachys* L. genus has a long and rich history of use in traditional medicine, e.g., in Italy, Greece, Turkey, Kosovo, Serbia, Montenegro, Poland, Iran, China, Japan, India, Brazil and Peru, where it has been prepared in a form of essential oil, tea, infusion and dried plant parts administered orally or directly on the skin (Gören, 2014; Polat et al., 2015; Tomou et al., 2020). Plants belonging to the *Stachys* L. genus constitute a rich source of diverse metabolites exhibiting a wide spectrum of biological activity: antioxidant (Kukić et al., 2006; Jassbi et al., 2014; Tundis et al., 2015), anticancer (Foruzani et al., 2015; Barmoudeh et al., 2022; Slimani et al., 2023) and antimicrobial (Saeedi et al., 2008; Koutsaviti et al., 2011; Lazarević et al., 2013; Gizem et al., 2021). The chemical composition of *Stachys* species varies and depends strongly on the type of plant habitat and geographical location, the availability of water and nutrients or the stage of plant development (Kanjevac et al., 2023). Among the most biologically active secondary metabolites, there are polyphenols, iridoids, terpenoids, fatty acids, alkaloids, triterpenes and many others, represented by more than 200 metabolites (Bilušić, 2019; Tomou et al., 2020). However, there are only scarce data on *Stachys sylvatica* L. derived mostly from Balkan Peninsula region (Hajdari et al., 2012; Jassbi et al., 2014; Dimitrova et al., 2015; Bilušić, 2019; Apostolescu et al., 2023; Di Stasi et al., 2023).

The study aimed to characterize the qualitative and quantitative chemical composition of hydroethanolic extract (50% v/v) from the aerial parts of *S. sylvatica* L. collected in the flowering stage (SSE) growing in Almaty region (Southern Kazakhstan) obtained with the aid of ultrasonication. The biological activity of this extract, including anthelminthic, antimicrobial and anticancer properties, was also evaluated.

## 2 Materials and methods

### 2.1 The collection of plant material and extraction procedure

The aerial parts of *Stachys sylvatica* L. were collected at the flowering stage in July 2021 at its wild habitat near Zailiysky Alatau mountain (Almaty, Kazakhstan; 43°11′44.5″N 77°07′11.0″E). During the collection of the raw material, more than 5 kg was collected, which was further used for the identification and standardization of the plant. For the extraction experiment described below, 25 g of the crushed raw material was used. The plants were identified following the requirements of the State Pharmacopoeia of the Republic of Kazakhstan by Sitpaeva Gulnara Tokbergenovna, at the Laboratory of the Flora of Higher Plants of the Institute of Botany and Phytointroduction of the Committee of Forestry and Wildlife of the Ministry of Ecology, Geology and Natural Resources of the Republic of Kazakhstan (Voucher No. 01–05/309). After collection, following the Guideline on Good Agricultural and Collection Practice, all impurities such as other plants or soil were removed. Extraction procedure was conducted following patent (Mukhamedsadykova et al., 2023). The plant material was air-dried under the canopy in natural conditions at temperature ranging from 30°C to 35°C for several days and shredded. Voucher specimens were deposited in the Institute of Botany and Phytointroduction for replenishment of the herbarium fund of the main botanical garden. First, the aerial parts of *S. sylvatica* L. composed of flowers, leaves and stems were cut into 3–4 cm pieces. Then, 300 mL of 50% ethanol (50% v/v) was added to 25 g of crushed raw material placed in a 500 mL flask and put in an alcoholic (SOUS) ultrasonic extractor (KQ5200B; Kunshan Instruments Inc., China) for 25 min (room temperature, 40 kHz). The process was repeated three times with the same solvent. Then, to obtain a dry extract, the solvent was removed with the use of an evaporation process at 45°C for 8 h (Concentrator plus, Eppendorf). The extraction yield was equal to 24.93 ± 0.28 g/100 g DW.

### 2.2 Reverse phase high-performance liquid chromatographic method with photodiode array analysis (RP-HPLC/PDA)

In preparing the SSE for determination, it was necessary to purify it of potential interfering components (e.g. chlorophyll). First, 25 mg of the extract was dissolved in 2 mL of methanol-water mixture (1:1 v/v) and then, using a solid phase extraction system (SPE system J.T. Baker, United States) and 500 mg C18 micro-columns (J.T. Baker, United States), the tested extract was applied to the octadecyl BakerBond cartridges, that had previously been appropriately spiked and conditioned (10 mL of MeOH and 10 mL of the methanol-water mixture (1:1 v/v)). The extract was eluted with a methanol-water mixture (1:1 v/v) in portions of the eluent, directly into the receiver, which was a 10 mL analytical volumetric flask. The operating pressure of the device was −0.01 Mpa. The extract was then prepared for qualitative and quantitative analysis.

Qualitative and quantitative analysis using high-performance column chromatography coupled to a photodiode array detector was performed using a Agilent Technologies (Waldbronn, Germany) 1,100 Series liquid chromatograph equipped with an autosampler. Analysis of the extract was performed using three wavelengths: 254 nm, 280 nm and 325 nm, due to the diversity of metabolites (phenolic acids and derivatives as well as flavonoids and their derivatives). A chromatographic column Zorbax Eclipse XDB C8 column (150 × 4.6 mm I.D., dp = 5 μm) was used, which allowed the extract to be analysed in 30 min using the following eluent gradient: A–water +1% (v/v) acetic acid; B–acetonitrile (0% min–10% B; 0–10 min–10%–14%B; 10–25 min–14%–30% B; 25–30 min–30%–33% B) at a flow-rate of the mobile phase of 1 mL/min and using a fixed analysis temperature of 25°C. Due to the use of the autosampler, it was necessary to use a postrun time of 5 min for the data analysis. For identification purposes, a library of standard substances was created for the particular HPLC/DAD analysis method used in the study. These standards were also collected with consideration of the starting concentration–for each standard substance at 1 mg/10 mL of solution. Qualitative analysis was based on the chromatographic (peak areas, retention times of individual standard compounds) and spectroscopic (UV spectra) data. Quantitative analysis was carried out, taking into account a threefold analysis (n = 3) on three consecutive days of the extract tested. Quantitative analysis of selected and identified metabolites was prepared in methanol:water (6:4 v/v) for phenolic compounds and in methanol for flavonoids at five concentrations ranging from 0.02 to 0.1 mg/mL. Five-point curves were constructed and regression equations together with R2 values were calculated for each calibration curve. The repeatability of peak areas was determined by the evaluation of both SD and RSD parameters.

### 2.3 Applied high performance liquid chromatography coupled with mass spectrometry based detection fingerprinting (HPLC-ESI-QTOF-MS/MS)

The fingerprinting of the obtained extract was performed on a platform composed of HPLC chromatograph (HP1200 Series, Agilent Technologies, Santa Clara, CA, United States) composed of a degasser, an autosampler, a binary pump, and a PDA detector - with a QTOF-MS/MS mass detector (6500 Series, Agilent Technologies, Santa Clara, CA, United States). The chromatographic separation of metabolites was achieved in an optimized method and by applying a Zorbax Eclipse Plus RP-18 chromatographic column (150 mm × 2.1 mm, dp = 3.5 µm, Agilent Technologies, Santa Clara, CA, United States). The chromatograms were recorded in a gradient of solvents: acetonitrile with 0.1% formic acid (solvent B) and 0.1% aqueous solution of formic acid (solvent A) as follows: 0 min–1% of B, 10 min–20% of B, 15 min–40% B, 17–22 min–95% B, 22.1–25 min–1% of B. The run lasted 30 min. The flow rate was set as 0.2 mL/min, the temperature of thermostat at 20°C, UV detection as 210, 254, 280, 365 and 320 nm and the injection volume was 10 µL.

Mass spectrometer was operated in both positive and negative ion mode in the mass range of 40–1,300 u, with the temperature settings of 275°C and 325°C for gas and sheath gas, respectively, gas flows of 12 L/min, capillary voltage of 3000 V, nebulizer pressure of 35 psig, collision energies of 10 and 20 V, fragmentor voltage of 110 V, and skimmer voltage of 65 V. Qualitative Navigator (version B.10.00) and Agilent Mass Hunter Data Acquisition (version 10.1) programs produced by Agilent Technologies (Santa Clara, CA, United States) were used to acquire the spectra and process the recorded data.

### 2.4 Gas chromatography–mass spectrometry analysis (GC-MS)

The SSE was further extracted by chloroform to obtain the volatile fraction. The GC-MS analysis was carried out with the use of a Shimadzu GC-2010 Plus gas chromatograph, coupled with a QP 2010 Ultra mass detector. Volatile metabolites were separated on a ZB-5MS capillary column (Phenomenex) with a silica film thickness of 0.25 µm, column length of 30 m, and internal diameter of 0.25 mm. The initial oven temperature was 50°C, with a 3 min holding time, and then increased to 250°C at 5°C/min, and held for 15 min at 250°C. An injection temperature of 280°C was maintained, and helium (1 mL/min) was used as a carrier gas. The QP 2010 Ultra mass spectrometer worked in the electron ionization (EI) mode. Ionization energy was 70 eV, the scan rate was 0.2 s/scan, and the scan range was 40–500 amu. The temperature of the ion trap was 220°C, while the injection and interface temperature was 250°C. The injection volume was 1 µL. The sample was dosed in split mode (1:20). The retention indices (RI) of the volatile metabolites present in the chromatograms were calculated concerning the homologous series of n-alkanes (C6–C30). The identification of metabolites was made using computer spectral libraries (MassFinder 2.1, NIST 2011) as well as available literature data.

### 2.5 Anthelminthic activity–examination method

The extract was tested for its anthelmintic activity using a model of nematodes of the *Rhabditis* genus in accordance with a previously patented research procedure; patent number PL232918 (Bogucka-Kocka and Kołodziej, 2019). The extract was added to the nematode culture in five selected concentrations: 0.2 mg/mL, 1.1 mg/mL, 3.3 mg/mL, 5.5 mg/mL, 11.1 mg/mL. After a 24-h exposure of nematodes of the genus *Rhabditis* to the tested extract, their viability was assessed according to the previously described procedure (Dziduch et al., 2020; Bogucka-Kocka et al., 2022; Kołodziej et al., 2023). The control (CTRL NaCl) was a culture of nematodes suspended in 0.6% NaCl only. The experiment was conducted in five biological replicates. Each biological replicate was carried out in three technical replicates. Statistical analysis was performed using GraphPad v8.2.0 software using ANOVA.

### 2.6 Antibacterial and antifungal activity

#### 2.6.1 Microbroth dilution method

The MIC (minimum inhibitory concentration) of SSE was evaluated with the use of the microbroth dilution method. Briefly, SSE was two-fold diluted in sterile Mueller-Hinton Broth (MHB; Biorad, Hercules, CA, United States) in a 96-well microtiter plate (NUNC, Rochester, New York, United States) to obtain a concentration ranging from 16 to 0.0156 mg/mL. Then, a microbial suspension of a density of 0.5 MacFarland was 100-fold diluted in MHB and 2 µL was added to each well. The assay included positive and negative control, i.e., a well with microorganisms growing without the tested compound and sterile MHB only, respectively. The adequate viability of reference microorganisms was confirmed by plating the liquid culture followed by a colony count with macroscopic characteristics. The plates were incubated at 35°C ± 2°C for 18–24 h (Heraeus B6, Germany) and MIC values were red spectrophotometrically based on the absorbance measured at 600 nm wavelength (EL80, BioTek Instruments, VT, United States). Then, to establish the MBC (minimum bactericidal concentration), 5 µL from each well was plated on Mueller-Hinton Agar (MHA; Biorad, Hercules, CA, United States) and incubated for another 16–20 h.

First, the antimicrobial activity of SSE was determined against a basic panel of reference microorganisms: bacteria *Staphylococcus aureus* ATCC 29213, *Escherichia coli* ATCC 25922, *Pseudomonas aeruginosa* ATCC 27853 and yeast *Candida albicans* ATCC 10231. Then, based on the initial results the microbial spectrum was expanded by adding the following Gram-positive bacteria: *Staphylococcus aureus* ATCC BAA-1707, *Staphylococcus epidermidis* ATCC 12228, *Bacillus cereus* ATCC 10876, *Enterococcus faecium* ATCC 19434, *E. faecalis* ATCC 51299 and *E. faecalis* ATCC 29212. Among the tested reference strains, *S. aureus* ATCC BAA-1707 possessed an acquired type of resistance to β-lactam antibiotics (MRSA–methicillin-resistant *S. aureus*) and *E. faecalis* ATCC 51299 exhibited resistance to glycopeptides (VRE–vancomycin-resistant *Enterococcus*). Internal controls included reference antimicrobials, such as vancomycin, ciprofloxacin and nystatin. The MIC values for ciprofloxacin against *S. aureus* ATCC BAA-1707, *S. epidermidis* ATCC 12228, *B. cereus* ATCC 10876, *E. coli* ATCC 25922 and *P. aeruginosa* ATCC 27853 were 0.48, 0.12, 0.06, 0.004 and 0.48 μg/mL, respectively. For *E. faecalis* ATCC 29212, *E. faecalis* ATCC 51299 and *E. faecium* ATCC 19434, the MIC of vancomycin was equal to 1.96, 32 and 0.98 μg/mL, respectively, while the MIC of nystatin against *C. albicans* ATCC 10231 was 0.48 μg/mL. All bacterial reference strains used in this study were cultured on MHA or MHB while *C. albicans* was cultured with the use of the same microbiological media but with 2% addition of glucose. The experiment was carried out in triplicate and the results were presented as mode.

#### 2.6.2 Time-kill assay

Time-kill assay was conducted to determine the mode of action of SSE against a microorganism with the lowest MIC value–*Bacillus cereus* ATCC 10876. First, a 0.5 McFarland *B. cereus* suspension was diluted 100 times and 5 mL was placed in the sterile tubes. The extract was added to the tubes to obtain a subinhibitory concentration (0.25 mg/mL), minimal inhibitory concentration (0.5 mg/mL) and a concentration exceeding MIC four times (2 mg/mL). These tubes along with a control tube (bacterial suspension without the tested extract) were incubated at 35°C for 24 h with shaking at 250 rpm (Innova 40R, New Brunswick, Germany). The number of colony forming units in 1 mL of suspension (CFU/mL) was determined by plating 100 µL of decimal dilutions on MHA followed by incubation at 35°C for 16–18 h. The enumeration was performed in eight time points: directly after the preparation of bacterial suspension as well as after 1, 3, 6, 12, and 24 h of incubation. The experiment was carried out in triplicate and the results were presented as the mean with standard deviation of a log_10_ of CFU/mL. Bactericidal activity of the extract was defined as the reduction of bacterial load equal to or greater than 3 log_10_ CFU/mL in comparison to the untreated control, while bacteriostatic activity was defined when the reduction was lower than 3 log_10_ CFU/mL. Microbiological data were analyzed with the use of Graph Pad Prism (V. 6.0; Graph Pad Software Inc., San Diego, CA, United States). Time-kill assay results were presented as graph curves, while the statistical analysis of AUC (area under the curve) was conducted with the use of one-way ANOVA followed by Dunnett’s *post hoc* test.

#### 2.6.3 Spore germination inhibition assay

Spores of *B. cereus* ATCC 10876 were prepared according to previously published protocol (Rukayadi et al., 2009), with minor modification. First, a single *B. cereus* colony was streaked on MHA and incubated at 30°C for 14 days to induce spore formation. Then, the culture was suspended in sterile 0.85% NaCl, and after thorough vortexing, divided into 1 mL portions in the 1.5 mL tubes. The tubes were incubated at 60°C for 30 min to kill *B. cereus* vegetative cells. Then, the tubes were centrifuged at 13,000 rpm for 30 min at 4°C followed by washing with 1 mL of sterile 0.85% NaCl. The supernatant was discarded and the pellet was suspended in 1 mL of 0.85% NaCl. The centrifugation and washing steps were repeated four times. The spores were counted by preparing decimal solutions and plating using the spread-plate technique. The quality of the process was assessed by staining the spore suspension with the use of the Schaeffer-Fulton endospore staining method. Spores were stored at −70°C for further analysis.

### 2.7 Cytotoxicity

The microculture tetrazolium assay (MTT) used to evaluate the viability of the SSE against normal cells was performed as previously described (Świątek et al., 2023). Briefly, the monolayer of selected cells in 96-well plates was incubated with serial dilutions (2–0.001 mg/mL) of SSE in cell media for 24–72 h. Afterward, cell media were removed, cells were washed with PBS, MTT-supplemented medium was added, and incubation continued for 3 h. The precipitated formazan crystals were dissolved using SDS/DMF/PBS solvent, and absorbance was measured (540 and 620 nm) using Synergy H1 Multi-Mode Microplate Reader (BioTek Instruments, Inc. Winooski, Vermont, United States) with Gen5 software (ver. 3.09.07; BioTek Instruments, Inc.). Data were exported to GraphPad Prism (version 7.04, GraphPad Software, Inc., La Jolla, CA, United States) to calculate CC_50_ and/or CC_10_, i.e., the concentration of the SSE, which caused a 50% and 10% decrease in cell activity compared to the control (cells not treated with the extract). Values were presented as mean ± SD obtained from three replicates.

The cytotoxicity of the SSE by the MTT method was determined for the following normal cell lines: MRC-5 (human embryonic lung fibroblasts; ATCC, CCL-171) as well as VERO (monkey kidney epithelial cells; ATCC, CCL-81).

### 2.8 Antiviral activity

Antiviral activity was tested against Human Coronavirus 229E (HCoV-229E; ATCC, VR-740) propagated in MRC-5 and against Human Herpesvirus type 1 (HHV-1, ATCC, VR-260) propagated in VERO. Cell lines were cultured using Modified Eagle Medium (MEM; Corning, Tewksbury, MA, United States) supplemented with antibiotics (Penicillin-Streptomycin Solution, Corning) and fetal bovine serum (FBS, Corning)–10% (cell passaging) and 2% (cell maintenance and experiments). Phosphate buffered saline (PBS) and trypsin were bought from Corning, whereas MTT (3-(4,5-dimethylthiazol-2-yl)-2,5-diphenyltetrazolium bromide) and DMSO (dimethyl sulfoxide) from Sigma (Sigma-Aldrich, St. Louis, MO, United States). Incubation was carried out in a 5% CO_2_ atmosphere at 37°C (CO_2_ incubator, Panasonic Healthcare Co., Tokyo, Japan). Ribavirin, a standard antiviral substance, was acquired from Sigma. The SSE stock solution was prepared by dissolving in cell culture grade DMSO (PanReac Applichem). The stock solution of SSE was stored at 8°C until used.

To evaluate the antiviral potential, the selected cells (MRC-5 or VERO) were seeded into a 48-well plate and incubated overnight. Then, the cells were infected with HCoV-229E or HHV-1 in 100-fold CCID_50_/mL (CCID_50_–50% cell culture infectious dose), leaving at least two wells with non-infected cells (cell control), and incubated for 1 h. Then, the cells were washed with PBS and SSE in non-cytotoxic concentration was added and incubated until CPE (cytopathic effect) was observed in the virus control (virus-infected, non-treated cells). The plates were observed using an inverted microscope (CKX41, Olympus Corporation, Tokyo, Japan) and frozen (−76°C). After thawing, the samples collected from anti-HCoV-229E assays were subjected to RNA isolation (QIAamp Viral RNA Mini Kit, Cat.: 52904 QIAGEN GmbH, Hilden, Germany) while DNA was isolated (QIAamp DNA Mini Kit, Cat.: 51304 QIAGEN GmbH) from samples collected during anti-HHV-1 tests. Subsequently, the RNA isolates were subjected to one-step RT-qPCR (reverse transcription-quantitative polymerase chain reaction) amplification using iTaq Universal SYBR Green One-Step Kit (Cat.: 1725150, Bio-Rad Laboratories, Life Science Group, Hercules, CA, United States) and primers 229E-F (5′-CAT​ACT​ATC​AAC​CCA​TTC​AAC​AAG-3′), and 229E-R 5′-CAC​GGC​AAC​TGT​CAT​GTA​TT-3′) on the CFX96 thermal cycler (Bio-Rad Laboratories). The RT-qPCR parameters amplification were as follows: reverse transcription reaction (50°C, 10 min), polymerase activation (95°C, 1 min), cycling (40 repeats: denaturation (95°C, 10 s), annealing and synthesis (65°C, 30 s), fluorescence acquisition), and melting curve analysis (65°C–95°C, 0.5°C increment/5 s). The DNA isolates were subjected to qPCR amplification using SsoAdvanced Universal SYBR Green Supermix (Bio-Rad Laboratories) and primers (UL54F–5′CGCCAAGAAAATTTCATCGAG 3′, UL54R–5′ ACA​TCT​TGC​ACC​ACG​CCA​G 3′) on the CFX96 thermal cycler. The amplification procedure was as follows: initial polymerase activation (98°C, 3 min); cycling (40 repeats: DNA denaturation (95°C, 10 s), annealing and synthesis (60°C, 30 s), fluorescence acquisition); melting curve analysis (65°C–95°C). The HCoV-229E and HHV-1 viral loads in the tested samples were assessed in relation to virus control based on the relative quantity (ΔCq) method using CFX Manager™ Dx Software (Bio-Rad Laboratories). To evaluate the sensitivity of RT-qPCR and qPCR, dilutions (10, 100, and 1000-fold) of virus RNA isolate (HCoV-229E) or DNA isolate (HHV-1), respectively, were prepared and analysed.

### 2.9 Anticancer activity

The MTT assay (described in detail above) was used to examine the anticancer activity of the SSE against FaDu (human hypopharyngeal cancer; ATCC, HTB-43), H1HeLa (human cervical adenocarcinoma; ATCC, CRL-1958), and RKO (human colon cancer; ATCC, CRL-2577) cell lines as well as against two human malignant melanoma cell lines: A-375 (ATCC, CRL-1619) and G361 (ATCC, CRL-1424). Cell line A-375 was cultured in Dulbecco’s modified Eagle’s medium (DMEM; Corning, New York City). The G361 cell line was cultured in McCoy′s 5A Medium (Corning, New York City). All culture media were supplemented with 10% heat-inactivated FBS (PAN-Biotech, Aidenbach, Germany) and antibiotics: 100 U/mL penicillin, 100 μg/mL streptomycin and 0.25 μg/mL amphotericin B (Sigma-Aldrich, St. Louis, MO, United States). Cell culture was maintained at 37°C in a humidified atmosphere of 5% CO_2_.

## 3 Results

### 3.1 Compositional studies of the *Stachys sylvatica* L. extract

The content of selected polyphenols in SSE is presented in Table 1. RP-HPLC/PDA analysis showed that the major metabolite identified in SSE was chlorogenic acid (1.59 mg/g dry extract). Quantitative analysis also revealed a relatively high concentration of luteolin derivatives (0.43–0.66 mg/g dry extract), followed by multiple chlorogenic acid isomers (0.13–0.32 mg/g dry extract) and verbascoside (0.28 mg/g dry extract). Moreover, a lower concentration of p*-OH* benzoic acid and caffeic acid (0.11 and 0.06 mg/g dry extract, respectively) was found. Chromatograms obtained with the use of RP-HPLC/PDA are presented in Supplementary Figure S1.

**TABLE 1:** The content of selected polyphenols in *Stachys sylvatica* L. hydroethanolic extract obtained by ultrasonic-assisted extraction.

No.	Metabolite	Detection at 325/254 nm	mg/g dry extract	± SD/RSD
1	neochlorogenic acid	325	0.13	0.0/2.0
2	chlorogenic acid	325	1.59	0.0/0.1
3	cryptochlorogenic acid	325	0.30	0.0/1.1
4	caffeic acid	325	0.06	0.0/2.6
5	*p*-OH benzoic acid	254	0.11	0.0/2.5
6	verbascoside	325	0.28	0.0/0.2
7	isochlorogenic acid	325	0.32	0.0/0.2
8	luteolin *7-O-*glucoside	254	0.40	0.0/1.0
9	luteolin *7-O-*glucuronide	254	0.66	0.0/0.5
10	luteolin derivatives	254	0.43	0.0/0.1

SD–standard deviation; RSD–relative standard deviation.

**FIGURE 1:**
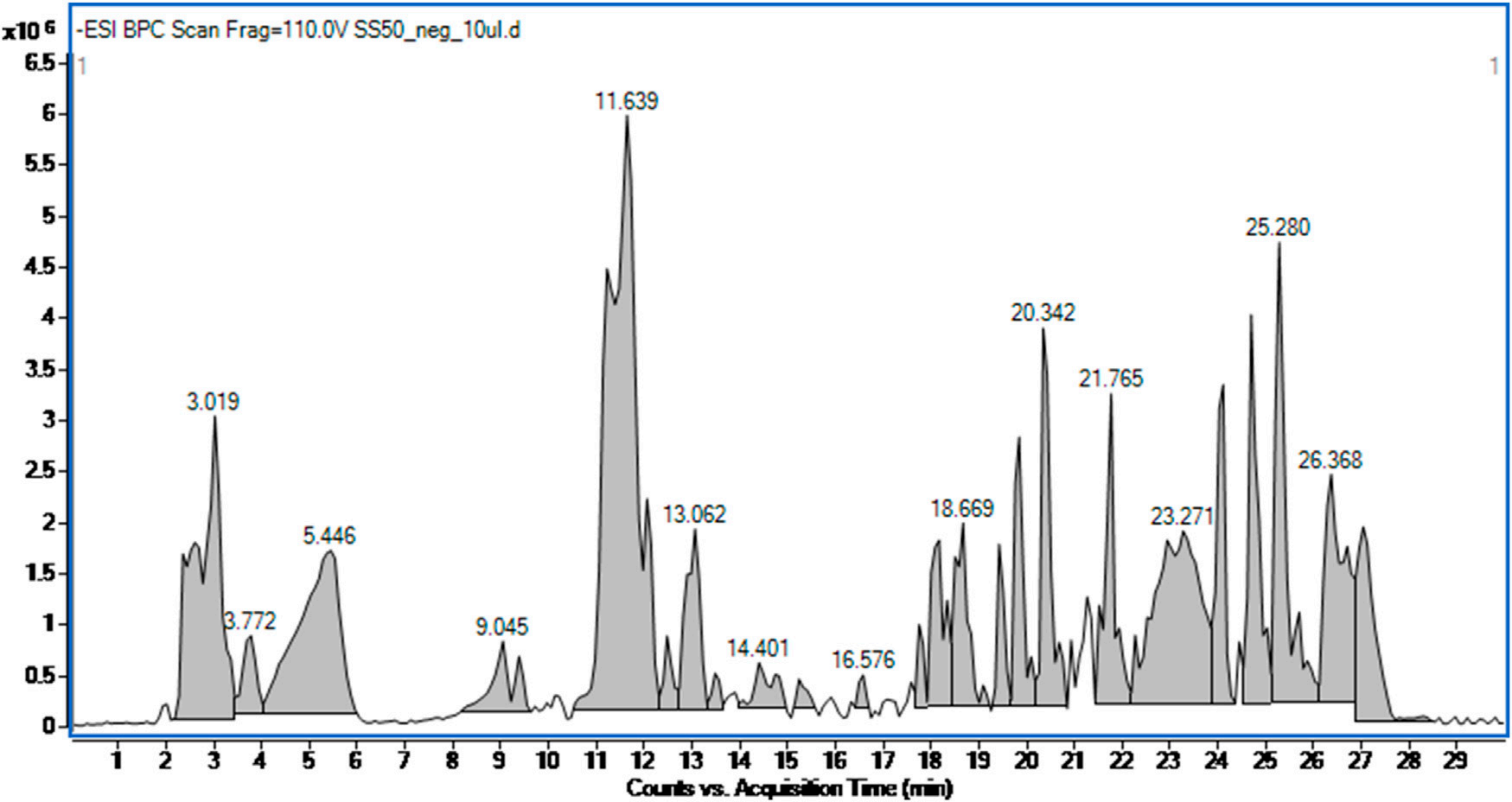
Total ion chromatogram of the *Stachys sylvatica* L. hydroethanolic extract obtained by ultrasonic-assisted extraction.

The applied high performance liquid chromatography coupled with mass spectrometry based detection of small molecules in the SSE led to the identification of 17 metabolites based on their high accuracy mass measurement, their MS/MS spectra (see Supplementary Table S1), retention times and the currently available scientific literature. According to the SSE fingerprinting, the main metabolites were phenolic acids, namely, chlorogenic acid and its isomers–cryptochlorogenic and neochlorogenic acid, phenolic metabolites such as flavonoids and their glycosides. The analysis also detected phenylpropanoids, e.g., verbascoside and its derivatives and verbasoside (decaffeoyl verbascoside) or harpagide belonging to iridoids (Figure 1; Table 2).

**TABLE 2:** The list of HPLC-ESI-QTOF-MS/MS tentatively identified metabolites in the *Stachys sylvatica* L. hydroethanolic extract obtained by ultrasonic-assisted extraction.

No.	Ion	Rt (min)	Formula	m/z (calc.)	m/z (exp.)	Δ (mmu)	RDB	MS/MS fragments	Metabolite
1	[M-H]^-^	8.9	C_20_H_30_O_12_	461.1664	461.1677	−2.7	6	419, 315, 161	Verbasoside
2	[M-H]^-^	9.4	C_16_H_18_O_9_	353.0878	353.0891	−3.66	8	191, 179	Neochlorogenic acid [1]^a^
3	[M + CH_3_COOH]^-^	10.1	C_40_H_53_O_24_	915.283	975.2975	−2.0	15	-	Isorhamnetin rhamnosylrutinoside-rhamnoside
4	[M-H]^-^	10.9	C_32_H_34_O_18_	705.1672	705.1701	−4.05	16	595, 513, 463, 403, 295	Kampferol triacetyl-pentoside-hexoside
5	[M-H]^-^	11.084	C_16_H_18_O_11_	385.0776	385.0806	−7.68	8	-	O-feruoylgalactarate
6	[M-H]^-^	11.1	C_16_H_18_O_9_	353.0878	353.0907	−8.17	8	191, 173	Chlorogenic acid [2]^a^
7	[M + HCOO]^-^	11.2	C_38_H_46_O_21_	837.2459	883.2550	−4.11	16	-	Acacetin
8	[M-H]^-^	13.06	C_16_H_18_O_9_	353.0878	353.0902	−6.76	8	191, 161	Cryptochlorogenic acid [3]^a^
9	[M-H]^-^	14.4	C_21_H_18_O_12_	461.0725	461.0739	−2.92	13	-	Luteolin glucuronide [9]^a^
10	[M-H]^-^	18.1	C_29_H_36_O_15_	623.1981	623.2023	−6.66	12	509, 461, 299, 161	Verbascoside [6]^a^
11	[M-H]^-^	18.25	C_21_H_20_O_11_	447.0933	447.0970	−8.29	12	285, 191	Luteolin glucoside [8]^a^
12	[M-H]^-^	18.35	C_29_H_18_ O_7_	477.0980	477.0981	−0.26	21	327, 285	Kaempferol hexoside
13	[M-H]^-^	18.4	C_29_H_36_O_15_	623.1981	623.2017	−5.7	12	509, 461, 299, 161	Isoverbascoside
14	[M-H]^-^	18.7	C_54_H_46_O_15_	933.2764	933.2794	−3.22	32	623, 463, 309	Verbascoside derivative
15	[M-H]^-^	18.75	C_33_H_40_O_20_	755.2040	755.2102	−8.18	14	679, 623, 161	Verbascoside derivative
16	[M-H]^-^	20.0	C_15_H_24_ O_9_	347.1348	347.1379	−9.03	4	-	Harpagide
17	[M-H]^-^	21.5	C_30_H_46_O_7_	517.3171	517.3186	−2.94	8	403, 357, 285, 187	Luteolin derivative [10]^a^

Ion–the type of ionization (+/−), RT–retention time, calc.–calculated, exp.–experimental, Δ–error of m/z measurement, RDB–the number of rings and double bonds.

^a^ metabolites identified and quantified by RP-HPLC/PDA method.

GC-MS analysis of the volatile metabolites showed mainly the presence of diterpenoids and fatty acids esters (Table 3). The most characteristic metabolites identified were fatty acid esters, however, the major was squalene, a linear triterpene synthesized by many plants as a biochemical precursor in the process of plant sterols biosynthesis. The relative percentage of this metabolite in the volatile fraction was almost 43%. The total ion chromatogram of the SSE and mass spectra of two unidentified diterpenoids are available in Supplementary Figures S2-S4.

**TABLE 3:** Volatile metabolites identified in *Stachys sylvatica* L. hydroethanolic extract obtained by ultrasonic-assisted extraction.

No.	Identified metabolite	RI_ex_	RI_lit_
1	Neophytadiene (isomer I)	1813	1807
2	Neophytadiene (isomer II)	1827	1830
3	Neophytadiene (isomer III)	1846	1847
4	Hexadecanoic acid	1950	1951
5	Isopropyl 14-methyl pentadecanoate	1959	1949
6	Unidentified diterpene^a^	1960	-
7	Octanoic acid dodecyl ester	2,151	2,177
8	Hexadecanoic acid tetradecyl ester	2,159	2,177
9	Nonanoic acid dodecyl ester	2,260	2,276
10	Unidentified diterpene of abietane type^a^	2,336	-
11	*n*-Tetracosane	2,393	2,407
12	*n*-Pentacosane	2,494	2,506
13	Squalene	2,893	2,914

RT–retention indices, exp–experimental, lit–literature.

^a^ mass spectra of unidentified diterpenoids are available in Supplementary Material.

### 3.2 Anthelminthic activity of the *Stachys sylvatica* L. extract

**FIGURE 2:**
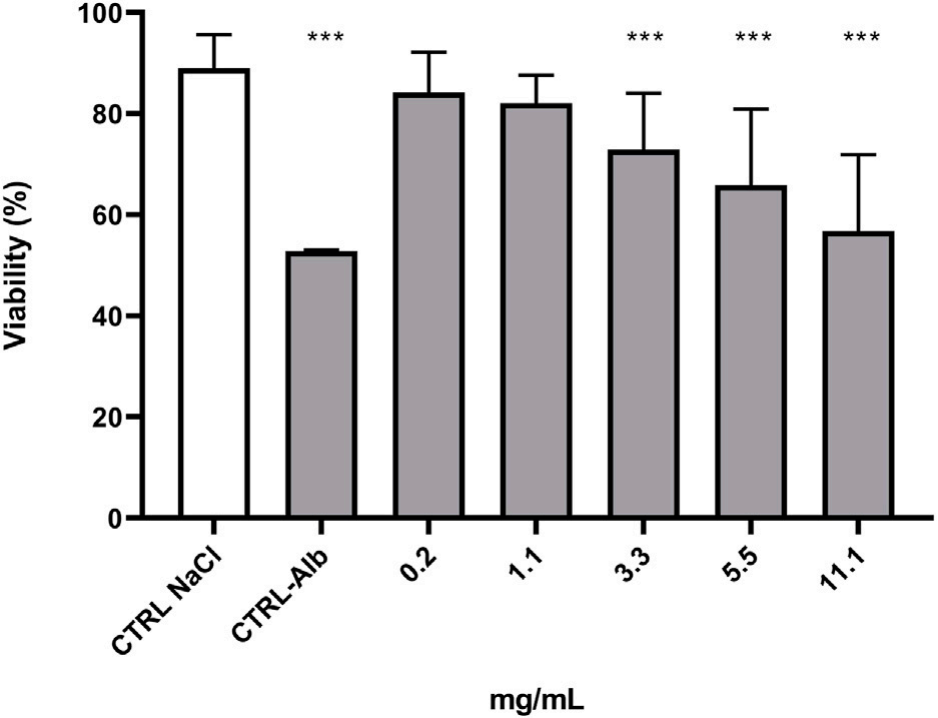
Viability (%) of *Rhabditis* sp. nematode cultures after 24 h of exposure to increasing concentrations of the *Stachys sylvatica* L. extract obtained by ultrasonic-assisted extraction. CTRL NaCl—control, CTRL-Alb (11.1 mg/mL)—positive control.

**FIGURE 3:**
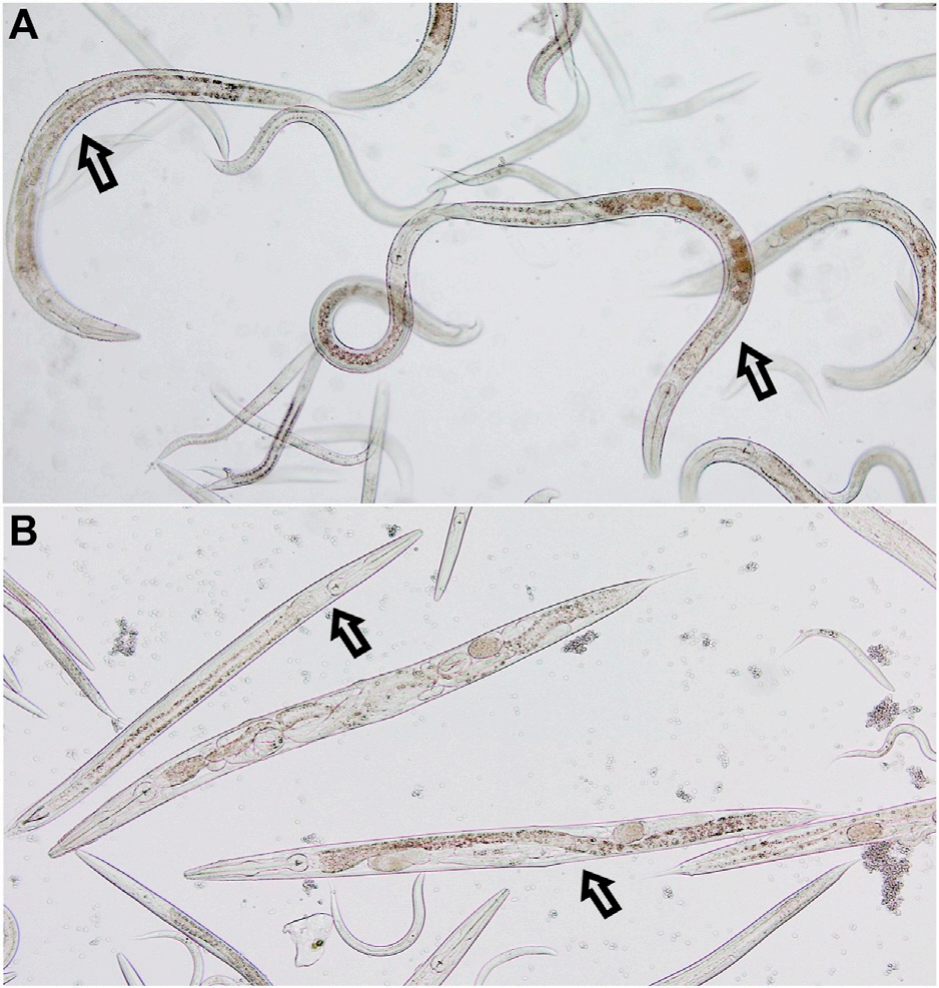
The culture of nematodes of the genus *Rhabditis*. **(A)** Control culture in 0.6% NaCl; **(B)** Culture after 24 h of exposure to the *Stachys sylvatica* L. extract obtained by ultrasonic-assisted extraction.

The anthelmintic activity of SSE was tested using nematodes of the genus *Rhabditis*. These nematodes belong to free-living organisms, but literature data indicate that they are also pathogenic organisms that can parasitize both humans and animals (Duarte et al., 2001; Teschner et al., 2014; Fadaei et al., 2019; Kołodziej et al., 2023). The experiments showed that the tested extract exhibited anthelmintic activity (Figure 2). At the tested concentrations, nematode viability was as follows: 84.21% for the concentration of 0.2 mg/mL, 82.08% for the concentration of 1.1 mg/mL, 72.88% for the concentration of 3.3 mg/mL, 65.84% for the concentration of 5.5 mg/mL and 56.76% for the concentration 11.1 mg/mL. Analysis showed that a significant reduction in *Rhabditis* sp. viability was obtained for the SSE concentration of 3.3 mg/mL or higher when compared to the control. The tested SSE showed anthelmintic activity comparable to that of albendazole in a concentration of 11.1 mg/mL (Ziaja-Sołtys et al., 2022); no statistically significant difference was demonstrated between reference drug vs. extract. Figure 3 presents exemplary photographs of nematodes used in this study. Upper part (A) shows *Rhabditis* sp. control culture in 0.6% NaCl, where the nematodes are alive and moving, while the lower part (B) shows the influence of SSE on their viability–non-moving and dead nematodes are marked with an arrow. Despite inhibition of nematodes viability at certain SSE concentrations, further research in this area is needed, among other things, to learn the mechanisms of action of the tested extract.

### 3.3 Antibacterial and antifungal activity of the *Stachys sylvatica* L. extract

The antimicrobial activity of SSE was tested against a basic panel of four microorganisms as well as additional six Gram-positive bacteria based on the initial results (Table 4). The microbroth dilution method showed that the antimicrobial activity was higher for Gram-positive bacteria than other tested microorganisms, i.e., Gram-negative rods as well as yeast, where in all cases the minimal inhibitory concentration was equal to 8 mg/mL. Among Gram-positive bacteria included in the study, the MIC range was from 0.5 to 2 mg/mL. In the case of *S. aureus* reference strains, the antimicrobial activity was slightly lower for the MRSA strain (methicillin-resistant *S. aureus* ATCC BAA-1707) with MIC equal to 2 mg/mL when compared with strain without an acquired type of resistance to β-lactam antibiotics. The opposite situation was observed in *Enterococcus* genus, where the VRE (vancomycin-resistant *E. faecalis* ATCC 51299) strain was characterized by a lower MIC value than susceptible strains (1 mg/mL vs. 2 mg/mL). The highest antibacterial activity was observed for *Bacillus cereus* ATCC 10876–an aerobic and spore-forming bacteria, where the MIC and MBC were equal to 0.5 mg/mL, which suggested its bactericidal mode of antimicrobial action.

**TABLE 4:** Antimicrobial activity of *Stachys sylvatica* L. hydroethanolic extract obtained by ultrasonic-assisted extraction.

Reference microorganism	MIC	MBC	MBC/MIC
	mg/mL	mg/mL	
Gram-positive bacteria			
*Staphylococcus aureus* ATCC 29213	1	2	2
*Staphylococcus aureus* ATCC BAA-1707	2	2	1
*Staphylococcus epidermidis* ATCC 12228	1	1	1
*Enterococcus faecium* ATCC 19434	1	4	4
*Enterococcus faecalis* ATCC 51299	1	4	4
*Enterococcus faecalis* ATCC 29212	2	4	2
*Bacillus cereus* ATCC 10876	0.5	0.5	1
Gram-negative bacteria			
*Escherichia coli* ATCC 25922	8	8	1
*Pseudomonas aeruginosa* ATCC 27853	8	8	1
Fungi (yeasts)	**MIC**	**MFC**	**MFC/MIC**
	mg/mL	mg/mL	
*Candida albicans* ATCC 10231	8	8	1

MIC–minimum inhibitory concentration; MBC–minimum bactericidal concentration; MFC–minimum fungicidal concentration.

**FIGURE 4:**
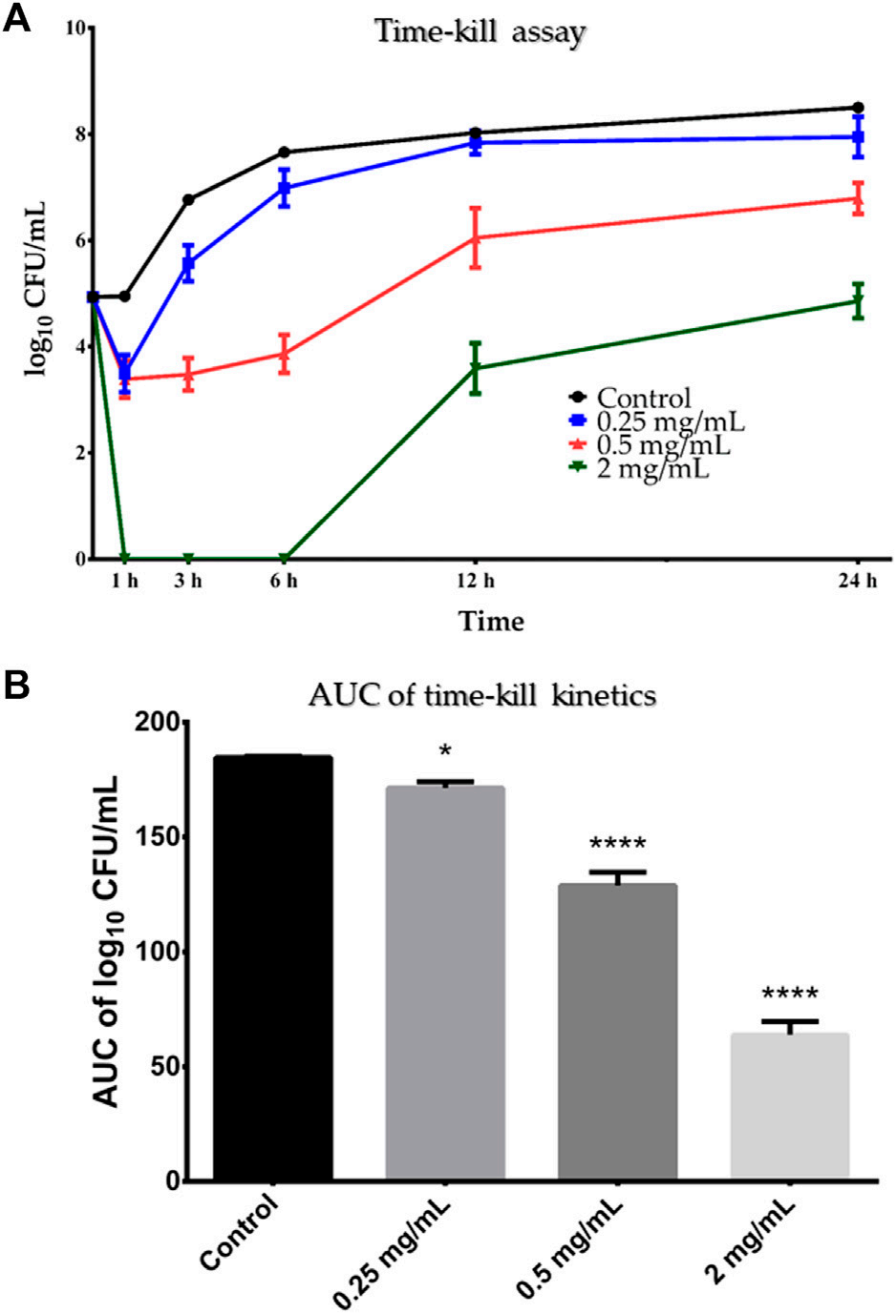
**(A)** Time-kill curve of *Stachys sylvatica* L. hydroethanolic extract obtained by ultrasonic-assisted extraction against *Bacillus cereus* ATCC 10876. A log_10_ of CFU/mL of *Bacillus cereus* ATCC 10876 at various time points in the presence of the *S. sylvatica* L. extract in concentration 0.25, 0.5 and 2 mg/mL in comparison to the growth control (without the inhibition agent). Values are expressed as mean ± standard deviation from three replicates. **(B)** AUC time-kill kinetics of *S. sylvatica* L. hydroethanolic extract against *B. cereus* ATCC 10876. Values are presented as mean ± standard deviation from three replicates. *p < 0.05; ****p < 0.0001 compared to the control. AUC–area under the curve.

Based on the results of the microbroth dilution method, *B. cereus* ATCC 10876 was chosen as the test microorganism for the time-kill assay, where it was incubated in the presence of three concentrations of SSE (0.5×MIC, 1×MIC and 4×MIC) and enumerated in certain time-points (Figure 4A). For the subinhibitory concentration of SSE (0.5×MIC; 0.25 mg/mL), minor growth inhibition of *B. cereus* cells was observed only after 1 h of incubation, which was then followed by a gradual rise in the number of viable cells comparable to that in the growth control. In the case of higher extract concentration equal to MIC–0.5 mg/mL–the number of viable *B. cereus* cells decreased after 1 h, remained relatively stable up to 6 h and then gradually raised reaching a comparable value at 24 h time point as in the growth control, testifying about the bacteriostatic mode of action of the tested extract. Only in the case of the highest applied extract concentration (2 mg/mL), there was a clear reduction of the number of viable cells starting from just 1 h after incubation up to 6 h of the experiment, where no growth was visible after plating. Then, after 6 h of incubation, the bacteria started to grow, resulting in the final reduction of bacterial load after 24 h equal to 3.3 log_10_ CFU/mL.

Statistical analysis of AUC showed that SSE acted on *B. cereus* in a dose-dependent manner (Figure 4B). Despite that all tested concentrations significantly reduced the number of viable cells of *B. cereus* ATCC 10876 in comparison to growth control, two higher concentrations–0.5 and 2 mg/mL–acted more effectively (*p* = 0.0001) than the subinhibitory concentration–0.25 mg/mL (*p* = 0.014).

The *Bacillus* genus is well known for its ability to produce spores–bacterial forms able to persist in unfavourable environmental conditions, often more resistant to tested compounds than vegetative cells. Therefore to determine whether SSE exhibits similar properties against spores as against vegetative cells of *B. cereus* ATCC 10876 we studied its sporicidal activity. The analysis showed that the minimum inhibitory concentration against spores was equal to 1 mg/mL, however, the minimum sporicidal concentration exceeded 16 mg/mL.

### 3.4 Cytotoxicity and antiviral activity of the *Stachys sylvatica* L. extract

Based on the 50% cytotoxic concentration (CC_50_) of the SSE, the cytotoxicity was classified as high (CC_50_ < 0.02 mg/mL), moderate (CC_50_: 0.021–0.2 mg/mL), weak (CC_50_: 0.21–0.5 mg/mL) or none (CC_50_ > 0.5 mg/mL), based on previously published literature (Geran, 1972; Łaska et al., 2019).

The cytotoxicity was determined towards two normal cell lines, namely, VERO (monkey kidney epithelial cells) and MRC-5 (human embryonic lung fibroblasts). After 72 h of incubation, the SSE showed moderate cytotoxicity towards MRC-5 with CC_50_ of 0.0891 ± 0.014 mg/mL and was not cytotoxic to VERO (CC_50_ of 0.810 ± 0.013 mg/mL).

**FIGURE 5:**
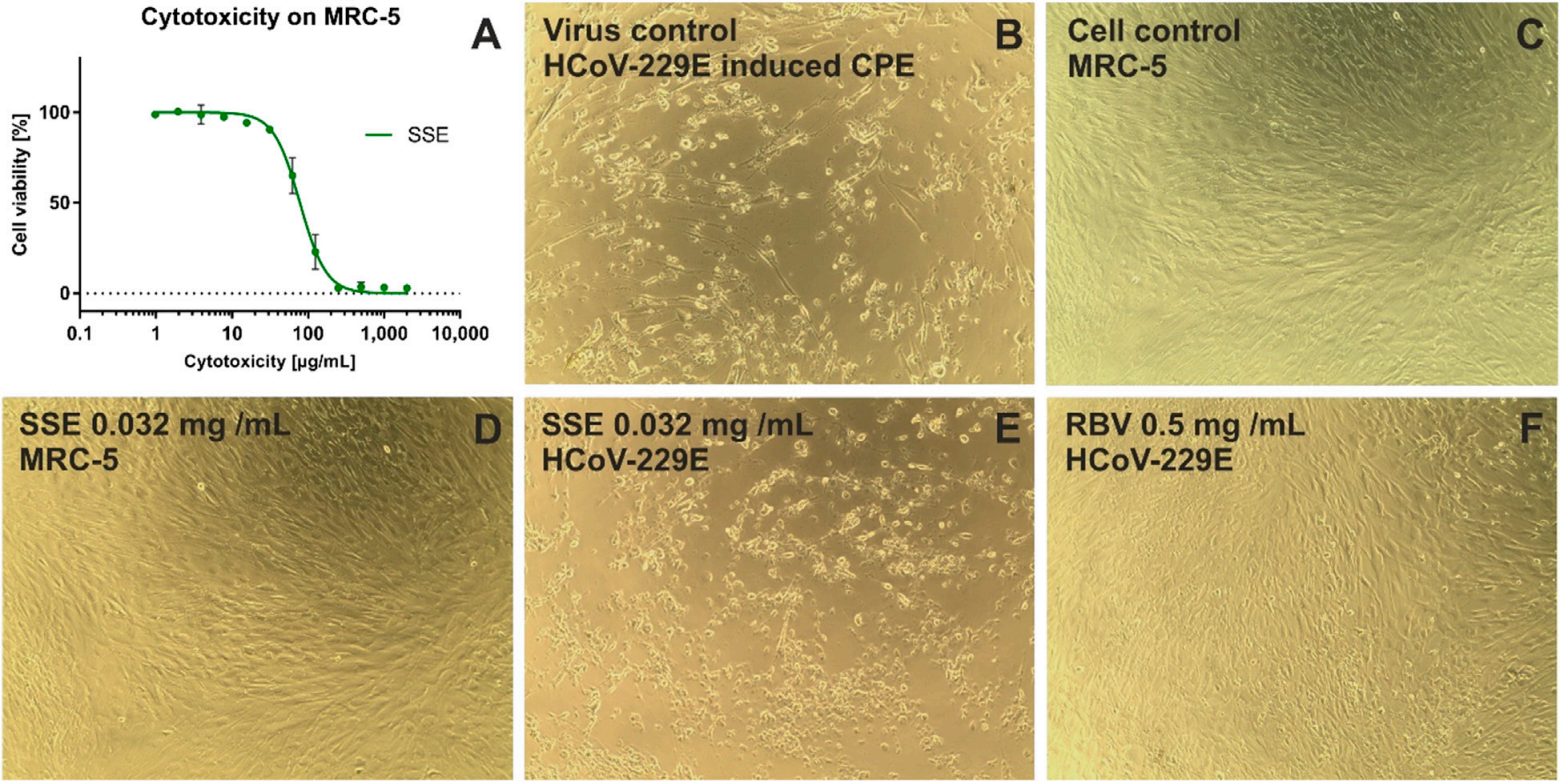
The cytotoxicity against MRC-5 and antiviral activity against HCoV-229E of *Stachys sylvatica* L. hydroethanolic extract obtained by ultrasonic-assisted extraction. **(A)** dose-response influence of SSE on MRC-5; **(B)** HCoV-229E induced cytopathic effect, virus control; **(C)** non-infected MRC-5 cells, cell control; **(D)** influence of SSE 0.032 mg/mL on MRC-5; **(E)** influence of SSE 0.032 mg/mL on HCoV-229E-infected MRC-5; **(F)** influence of ribavirin (RBV) 0.5 mg/mL on HCoV-229E-infected MRC-5.

**FIGURE 6:**
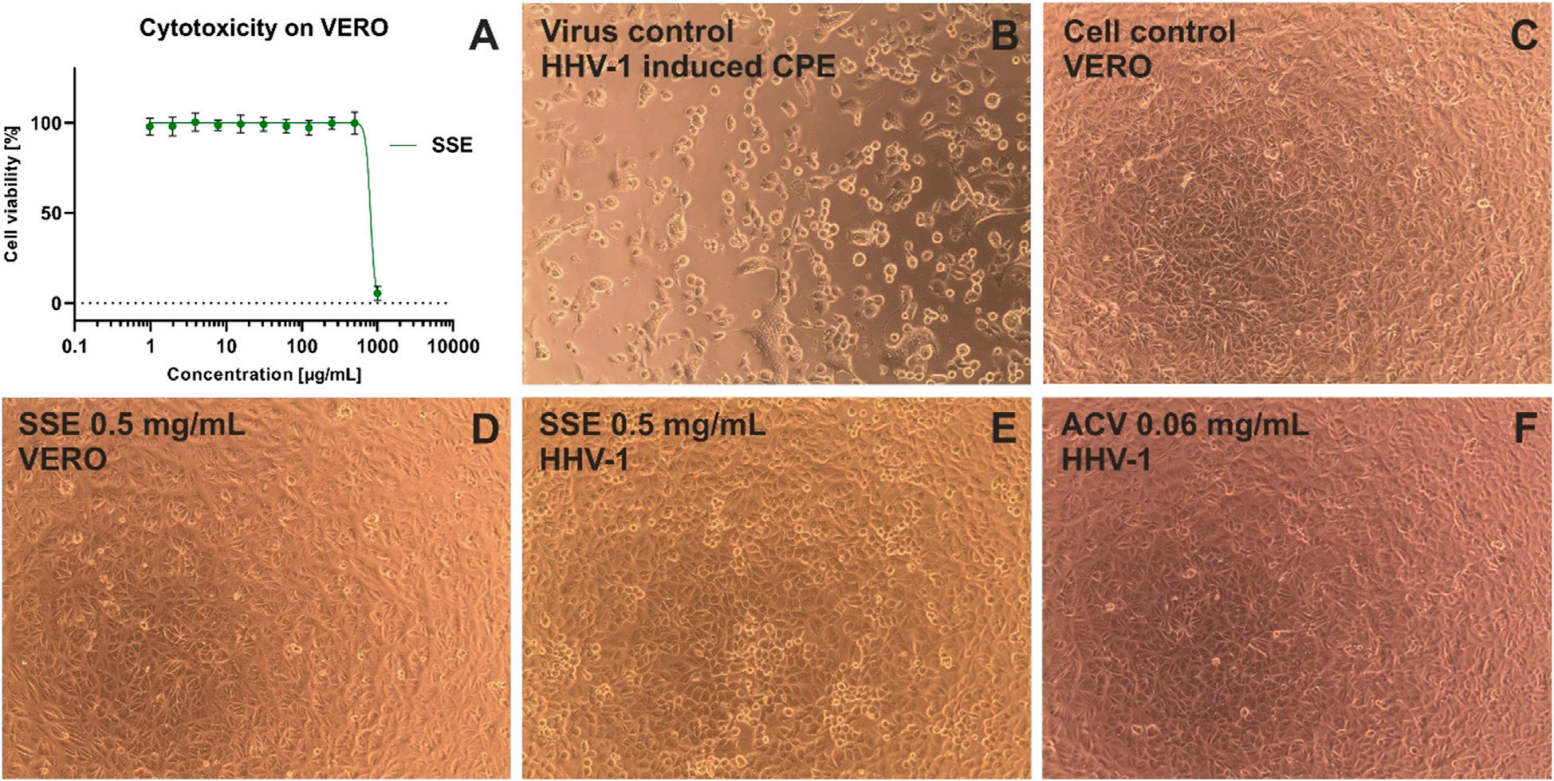
The cytotoxicity against VERO and antiviral activity against HHV-1 of Stachys sylvatica L. hydroethanolic extract obtained by ultrasonic-assisted extraction. **(A)** dose-response influence of SSE on VERO; **(B)** HHV-1 induced cytopathic effect, virus control; **(C)** non-infected VERO, cell control; **(D)** influence of SSE 0.5 mg/mL on VERO; **(E)** influence of SSE 0.5 mg/mL on HHV-1-infected VERO; **(F)** influence of acyclovir (ACV) 0.06 mg/mL on HHV-1-infected VERO.

**FIGURE 7:**
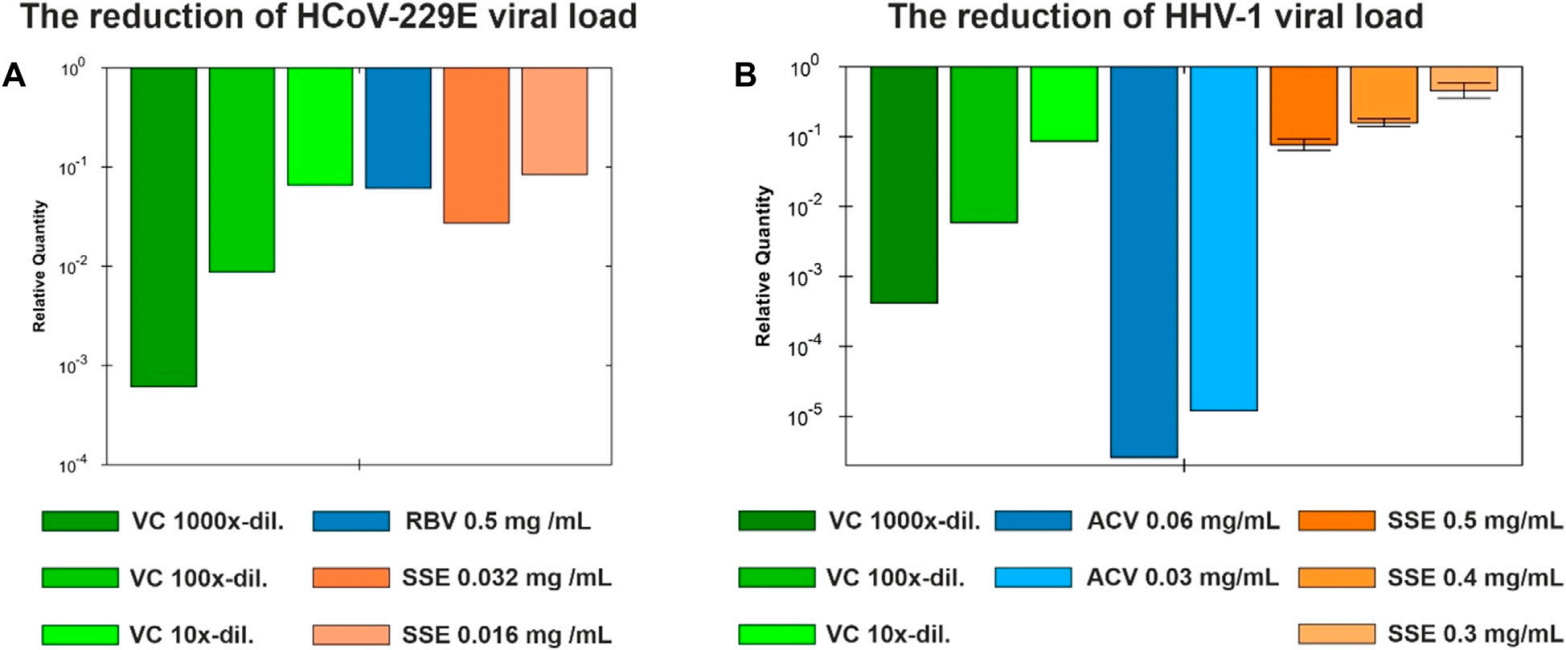
The viral load reduction under the influence of Stachys sylvatica L. hydroethanolic extract obtained by ultrasonic ultrasonic-assisted extraction. **(A)** The reduction of HCoV-229E viral load in MRC-5; **(B)** The reduction of HHV-1 viral load in VERO.

Based on the dose-response curves presented in Figure 5A and Figure 6A, the maximum non-toxic concentrations used in antiviral studies were estimated to be 0.032 mg/mL in MRC-5 and 0.5 mg/mL in VERO: In these non-toxic concentrations no cytopathic effects were observed in MRC-5 (Figures 5C, D) and in VERO (Figures 6C, D). The incubation of HCoV-229E-infected MRC-5 with SSE 0.032 mg/mL (Figure 5E) did not reduce the formation of CPE when compared with the virus control (Figure 5B). Ribavirin, a broad-spectrum antiviral, managed to noticeably reduce the formation of CPE (Figure 5F). Subsequent RT-qPCR analysis showed (Figure 7A) that despite the lack of influence on CPE formation, the SSE 0.032 mg/mL managed to reduce the viral load of HCoV-229E by 1.56 log, compared to the virus control. The antiviral effect showed a dose-response effect, and SSE at 0.016 mg/mL reduced the HCoV-229E viral load by 1.08 log. Interestingly, the ribavirin showed a reduction of only 1.21 log. Thus, the SSE showed low antiviral activity against HCoV-229E replicating in MRC-5, but comparable to ribavirin.

Interestingly, the incubation of HHV-1-infected VERO with SSE (0.5 mg/mL) (Figure 6E) noticeably reduced the formation of CPE when compared with the virus control (Figure 6B). The effect was dose-dependent, and at lower concentrations of SSE (0.3 and 0.4 mg/mL), the inhibition of CPE was less profound (data not shown). Following the qPCR analysis (Figure 7B), it was found that the SSE in the concentrations of 0.3, 0.4, and 0.5 mg/mL reduced the HHV-1 viral load by 0.34, 0.8, and 1.11 log, respectively. Acyclovir, a reference antiviral drug against HHV-1, at concentrations of 0.03 and 0.06 mg/mL, prevented the development of CPE in virus-infected VERO (Figure 6F) and reduced HHV-1 viral load by 4.92 and 5.59 log, respectively (Figure 7B). Thus, the SSE showed good protection against the cytopathic effect induced by HHV-1.

### 3.5 Anticancer activity

Preliminary evaluation of anticancer activity showed that the SSE exerted concentration- and time-dependent effect. This extract was moderately cytotoxic after 72 h incubation towards human cervical adenocarcinoma (H1HeLa) cells (CC_50_ of 0.0127 mg/mL), and weakly cytotoxic (0.21 < CC_50_ < 0.5 mg/mL) to human hypopharyngeal cancer (FaDu) and human colon cancer (RKO) cells (Table 5). After 24 h extract showed weak cytotoxicity on FaDu and H1HeLa and no cytotoxic effect (CC_50_ > 0.5 mg/mL) on RKO. After 24 h of incubation, the SSE exhibited weak cytotoxicity or no cytotoxicity against human melanoma cells, G361 and A375, respectively.

**TABLE 5:** The cytotoxicity of *Stachys sylvatica* L. hydroethanolic extract obtained by ultrasonic assisted extraction against normal and cancer cells.

Cell line		50% cytotoxic concentration (CC_50_, mg/mL)*	
		24 h incubation	72 h incubation
Non-cancer	VERO	0.892 ± 0.006	0.810 ± 0.013
	MRC-5	nd	0.0891 ± 0.014
Cancer	FaDu	0.343 ± 0.033	0.206 ± 0.011
	H1HeLa	0.231 ± 0.027	0.127 ± 0.009
	RKO	>0.5	0.252 ± 0.004
	G361	0.484 ± 0.003	nd
	A375	>0.5	nd

nd–not determined; mean ± SD.

## 4 Discussion

Based on the scientific literature data the genus *Stachys* L. can be perceived as a promising one with a high load of secondary metabolites of different types with proven pharmacological properties. As shown by the recent studies focusing on the phytochemical composition of *Stachys* L. genus, there is a noticeable heterogeneity of obtained results, depending on various factors such as geographical region, collection season and part of plant (Tomou et al., 2020). Nevertheless, studies have shown that flavonoids, iridoids, fatty acids and phenolic acids constitute the main plant metabolites present in extracts prepared from *Stachys* spp. (Tundis et al., 2014). Although several plant species belonging to this genus have been broadly studied (Tomou et al., 2020), there are only some phytochemical and biological/pharmacological investigations concerning *S. sylvatica* L. Data included in this paper presents for the first time the chemical composition and bioactivity of the hydroethanolic extract (50% v/v) obtained by ultrasonication from the flowering aerial parts of *S. sylvatica* L. collected in Almaty region (Southern Kazakhstan).

Previously other authors showed the presence of a rich variety of metabolites in *Stachys sylvatica* L. extracts. The chloroform, chloroform-methanol and methanolic extracts prepared from aerial parts of *S. sylvatica* L. collected in Tuscan-Emilian Apenine (Italy) were investigated, followed by the identification of 23 metabolites belonging to the phenolic derivatives, glycosylated aliphatic alcohols, glycosylated phenylpropanoids, flavonoids, lignans, ionones and carboxylic acids (Di Stasi et al., 2023). In other studies concerning *S. sylvatica* L. growing in Romania the hydromethanolic extracts (80% v/v) obtained from flowers, leaves and stems were analyzed separately. These extracts were rich in phenolic metabolites, including chlorogenic acid. The content of polyphenols in flowers, leaves and stems differed greatly, being 2119.69, 333.25 and 113.38 μg/g, respectively (Apostolescu et al., 2023; Di Stasi et al., 2023).

Our study showed that chlorogenic acid is the main metabolite in SSE (1.59 mg/g dry extract), which is consistent with other studies investigating different species belonging to the genus *Stachys* L, namely, *S. byzantina*, *S. inflata*, *S. lavandulifolia*, *S. annua* and *S. tmolea*. (Elfalleh et al., 2019; Bahadori et al., 2020; Bursal et al., 2020). For instance, this phenolic acid was found to be the dominant metabolite in the hydroethanolic extract (70% v/v) from aerial parts of *S. sylvatica* L. growing in Romania, where its concentration was 5.55 mg/g dry extract. The SSE was also found to contain high amounts of luteolin derivatives (1.49 mg/g dry extract), including luteolin 7-*O*-glucoside and luteolin 7-*O*-glucuronide (Benedec et al., 2023). According to the review of Tomou et al., this flavone and its derivatives were reported to be identified in aerial parts or leaves of several *Stachys* species, e.g., *S. aegyptiaca* Pers., *S. chrysantha* Boiss. and Heldr., *S. annua* L. or various subspecies of *S. wainsonii* (Tomou et al., 2020). As presented elsewhere (Lachowicz-Wiśniewska et al., 2022), one of the major metabolite of *S. palustris* L. leaves collected in Poland were luteolin 6-C-galactoside (462.91 mg/100 g dry matter) and luteolin-6-C-glucoside (267.68 mg/100 g dry matter). The phenylethanoid glycosides, including verbascoside, were found to occur commonly in various *Stachys* L. species, both in aerial parts and roots. The verbascoside content in the SSE was calculated to be 0.28 mg/g dry extract. It is worth noting that the extract was also rich in verbasoside and harpagide belonging to iridoids, as well as in organic acids, alkaloids and terpenoids (Tundis et al., 2015; Tomou et al., 2020).

According to quantitative analysis, dominant metabolites in the SSE were chlorogenic acid (together with its derivatives), luteolin derivatives and verbascoside. The chemical profile obtained for the investigated SSE partially differs from other *S. sylvatica* L. extracts, where all investigated samples were derived from plants growing in Europe (Italy or Romania). Phenolcarboxylic acids, mostly chlorogenic acid, caffeic acid and rosmarinic acid, are often found metabolites in *S. sylvatica* L. (Apostolescu et al., 2023; Benedec et al., 2023; Di Stasi et al., 2023). While in our extract chlorogenic acid was found, no remaining two acids were identified. Additionally, quantitative analysis revealed that *S. sylvatica* L. collected in Kazakhstan is rich in numerous chlorogenic acid derivatives, e.g., isochlorogenic acid or cryptochlorogenic acid, rarely reported in samples collected in other parts of the world (Benedec et al., 2023; Di Stasi et al., 2023). However, it is worth mentioning that chlorogenic acid derivatives were reported in research including other *Stachys* species, e.g., isochlorogenic acid was found in *S. officinalis* L. (Vundać et al., 2005; Kanjevac et al., 2023), while neochlorogenic and cryptochlorogenic acids in *S. palustris* L. (Kartsev et al., 1994). Similarly, we detected multiple luteolin derivatives, which were not detected in other studies concerning *S. sylvatica* L. (Benedec et al., 2023; Di Stasi et al., 2023).

Many studies focus on the chemical composition of the essential oil prepared from *S. sylvatica* or the content of volatile metabolites. Similarly, as in the case of chemical composition from the extracts prepared from the aerial parts of the plant, the results on the chemical composition of essential oils vary. Our studies showed that the SSE volatile fraction is composed mainly of diterpenoids and fatty acid esters. The obtained results are confirmed in the literature. Genus *Stachys* is a rich source of diterpenes mainly belonging to the group of kauranes, neoclerodanes, labdanes, and phytanes (Piozzi and Bruno, 2011). Among other volatile metabolites identified, the major were γ-muurolene, phytol, benzaldehyde (Dimitrova et al., 2015) or α-pinene, β-pinene and germacrene-D (Hajdari et al., 2012).

Currently, a lot of efforts are being put into the development of new anthelmintics for treating nematodes both in animals and humans, mostly due to restricted therapeutic options available, often additionally limited by growing resistance (Nixon et al., 2020). Our study showed anthelminthic activity of SSE against the nematodes from the genus *Rhabditis*–the extract decreased the viability of the organisms at the concentration 3.3 mg/mL or higher. Additionally, the extract in concentration 11.1 mg/mL showed an antiparasitic effect comparable to that of albendazole, a broad-spectrum anthelmintic applied in the treatment of parasitic infections (Chai et al., 2021). There are scarce data concerning the antiparasitic activity of *Stachys* L. genus. As reported elsewhere, *S. palutris* L. methanolic extract prepared from the plants collected in the United States at a concentration 50 mg/mL showed 72.9% ± 1.8% of inhibition on egg hatching in pathogenic nematode *Haemonchus contortus* (Acharya et al., 2014). Similarly, Barati et al. showed a significant decrease in the number of *Giardia lamblia* cysts under the influence of *S. lavandulifolia* n-hexane and water extracts prepared from the leaves of the plant (Barati et al., 2017), while Alanazi et al. found a relevant activity of its methanolic extract against *Toxoplasma gondii* (Alanazi et al., 2023)^40^.

It is generally accepted that polyphenols are a very important group of plant secondary metabolites with a wide spectrum of proven biological activities (Naveed et al., 2018; Muruganathan et al., 2022). Chlorogenic acid as well as luteolin and its derivatives are considered to be the important polyphenolic bioactive metabolites, responsible for antibacterial activity (Gupta et al., 2022; Le et al., 2022). Similarly, verbascoside was also reported to exert antibacterial properties (Tian et al., 2021). Despite multiple studies documenting chlorogenic acid’s wide spectrum of biological activity, further research is needed to determine whether it is an active marker in the case of the SSE studied here. As shown before, a marker compound, i.e., compound useful in terms of technological processes or research purposes, is often not the biologically active plant metabolite and even its concentration in the extract does not necessarily correlate with biological activity or therapeutic efficacy (Eisner, 2001; Ruiz et al., 2016; Heinrich et al., 2022).

The results presented in this paper on the antimicrobial activity of SSE indicated higher activity towards Gram-positive bacteria (MIC = 0.5–1 mg/mL) than Gram-negative bacteria (MIC = 8 mg/mL), exerting bactericidal effect. Similar observations were made by other authors, both for *S. sylvatica* L. as well as for other *Stachys* L. species, including *S. spruneri* Boiss., *S. spreitzenhoferi* Heldr. and *S. byzantina* C. Koch. (Saeedi et al., 2008; Koutsaviti et al., 2011; Stegarus et al., 2021; Napolitano et al., 2022). The sensitivity to SSE of yeast reference strain *C. albicans* ATCC 10231 used in the present study (MIC = 8 mg/mL) was comparable to that of Gram-negative bacteria. It is worth mentioning that noteworthy antibacterial or antifungal activity could be assigned to the plant extracts showing MIC ≤1 mg/mL (Ríos and Recio, 2005). In our studies, the lowest MIC value (0.5 mg/mL) was observed for *B. cereus* ATCC 10876, a Gram-positive and spore-forming bacterium responsible for food poisoning (Jovanovic et al., 2021). Also, Dulger et al. proved a significant antimicrobial activity of endemic *Stachys* L. species growing in Turkey against *B. cereus*, which reflects on significant potential against this bacterium (Dulger et al., 2005; Dulger and Aki, 2009). In the case of the highest extract concentration applied in the time-kill assay (2 mg/mL), there was a noticeable rise in the number of viable *B. cereus* cells after the initial period of the experiment (up to 6 h) where no growth was observed after plating. This situation may be due to the spore-forming abilities of *B. cereus*, enabling the bacterium to protect itself from the harmful action of the inhibition agent and allowing it to regrow after a certain period. This hypothesis was confirmed by the minimal sporicidal concentration obtained in our study exceeding 16 mg/mL, which was considerably higher than the concentration needed to inhibit the growth of *B. cereus* ATCC 10876 vegetative cells.

Antiviral studies showed that the SSE did not influence the formation of HCoV-229-induced cytopathic effect in MRC-5, but reduced the viral load by 1.56 log at maximum possible non-toxic concentration, i.e. 0.032 mg/mL. Additionally, the SSE noticeably reduced the formation of HHV-1 induced CPE in VERO at the highest tested concentration (0.5 mg/mL). Higher concentrations of the SSE could not be investigated for the antiviral activity assessment due to the limitations resulting from the toxicity towards normal cell lines. Although the antiviral activity of *S. sylvatica* L. has not been yet reported in the literature, selected metabolites of *S. lavandulifolia* Vahl. were assessed in terms of their potential antiviral activity against SARS-CoV-2 by molecular docking. It was found that among others, chlorogenic acid can be regarded as an anti-SARS-CoV-2 agent with potent protease inhibitory activity (Kılınç et al., 2022).

According to preliminary data presented in this paper, the SSE was moderately cytotoxic towards human cervical adenocarcinoma (H1HeLa) cells (CC_50_ of 0.127 mg/mL after 72 h). Although anticancer activity of *S. sylvatica* L. has not been reported before, there are some data concerning other *Stachys* L. species. Háznagy-Radnai et al. described that hydromethanolic extracts (80% v/v) from *S. recta* L. (stems), *S. palustris* L. (stems, folium, and flowers), *S. germanica* L. (flowers), and *S. byzantina* K. Koch (stems) showed variable anticancer effects against A431 (skin epidermoid carcinoma), HeLa (cervical adenocarcinoma) and MCF-7 (breast adenocarcinoma), with inhibition of cancer cell growth at 10 μg/mL, ranging from <25 to 55.4% (Háznagy-Radnai et al., 2008). Extract from the stem of *S. recta* L. showed the highest inhibition. Khanavi et al. showed that hydromethanolic extracts (80% v/v) from *S. laxa* Boiss. and Buhse., *S. subaphylla* Rech. F., *S. trinervis* Aitch. and Hemsl., and *S. turcomanica* Trautv exert moderate to no cytotoxic effects towards HT-29 and Caco-2 (colon adenocarcinoma), T47D (breast carcinoma), and NIH 3T3 (Swiss embryo fibroblast) (Khanavi et al., 2012). However, the fractionation of those extracts using chloroform, ethyl acetate, and methanol, produced some fractions showing moderate cytotoxicity. Thus, further studies on anticancer potential of *S. sylvatica* L. should be continued.

The *in vivo* studies on a model of experimental animals confirmed possible applications of hydroalcoholic *S. sylvatica* L. extracts. For instance, it was proved that the antioxidative and anti-inflammatory properties of the extract connected mainly with the presence of iridoids, flavonoids and sesquiterpenes improved the symptoms of polycystic ovary syndrome in rats (Alanazi et al., 2023). The promising results concerning other *Stachys* L. taxa were also obtained in the studies conducted on mice, e.g., certain fractions of *S. riederi* var. *japonica* (Miq.) H. Hara exhibited antidiabetic activity (Saravanakumar et al., 2021), while *S. lavandulifolia* Vahl.–anxiolytic and antidepressant properties (Modarresi et al., 2020).

Plants have been used in traditional medicine all over the world due to their rich application in the treatment of many diseases, ranging from mild health disorders to life-threatening epidemics (Silva and Fernandes Junior, 2010). Thanks to their valuable properties, knowledge on various plant species have been passed through generations making it possible to study their biological activity in the present times. Currently plethora of scientific studies confirm their biological activity giving an insight into their possible applications in medicine (Gülçin et al., 2004; Tohma et al., 2016; Yapıcı et al., 2021). In our study we proved that *S. sylvatica* L., a traditional medicinal plant characterized by a high variety of applications in folk medicine, includes several metabolites with high biological activity, including antibacterial and anthelminthic properties.

This study has some limitations. Despite the genus *Stachys* is widely disseminated around the world, a plant material consisting of *S. sylvatica* L. used in this study was collected locally, thus its analysis does not reflect the features of general population. Additionally, there are other factors leading to the alteration of chemical composition of the plant, including environmental conditions and plant-related characteristics, such as age of the plant (Pant et al., 2021).

## 5 Conclusion

The hydroethanolic extract (50% v/v) obtained with the aid of ultrasonication from *S*. *sylvatica* L. aerial parts collected at the flowering stage in the Almaty region, Southern Kazakhstan can be regarded as a plant material rich in polyphenols, especially chlorogenic acid and its isomers as well as luteolin derivatives. Our research contributes to the current knowledge of *S. sylvatica* L. chemical composition and biological activity. *S. sylvatica* L. collected in Kazakhstan partially differs in terms of chemical composition from species collected in Southern Europe. A promising bioactivity of this extract, including anthelmintic properties was described for the first time, and therefore can be regarded as a prerequisite for further *in vitro* and *in vivo* studies.

## Data Availability

The original contributions presented in the study are included in the article/Supplementary Material, further inquiries can be directed to the corresponding author.
