# Supplementary material for: Anthelminthic and antimicrobial effects of hedge woundwort (Stachys sylvatica L.) growing in Southern Kazakhstan
Source: Front Pharmacol. 2024 May 6;15:1386509. doi: 10.3389/fphar.2024.1386509 (PMC11102979; doi:10.3389/fphar.2024.1386509)
Supplement: Supplementary file 1 [file DataSheet1.docx]

Supplementary Material

Biological activity studies of *Stachys sylvatica* L. growing in Southern Kazakhstan revealed its promising anthelminthic effect

**Aigerim Z. Mukhamedsadykova, Martyna Kasela*, Kaldanay K. Kozhanova, Zuriyadda B. Sakipova, Wirginia Kukuła-Koch, Aleksandra Józefczyk, Łukasz Świątek, Barbara Rajtar, Magdalena Iwan, Przemysław Kołodziej, Agnieszka Ludwiczuk, Gulnara M. Kadyrbayeva, Gulnur N. Kuntubek, Aliya S. Mamatova, Anna Bogucka-Kocka, Anna Malm**

**Correspondence:** Martyna Kasela: [martyna.kasela@umlub.pl](mailto:martyna.kasela@umlub.pl)

# Supplementary Figures and Tables

## Supplementary Figures


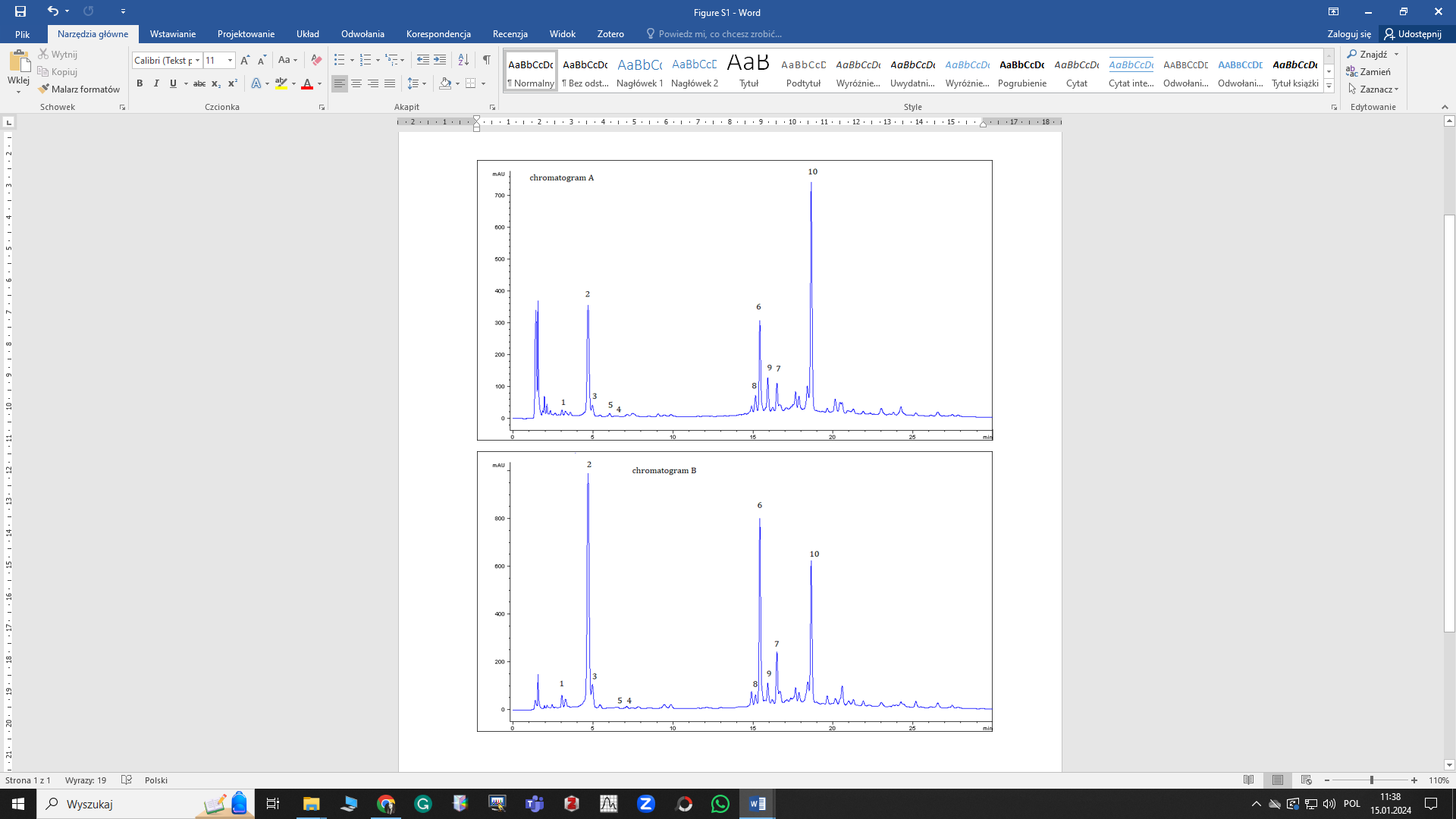


**Supplementary Figure S1.** Chromatograms obtained with the use of RP-HPLC/PDA. Chromatogram A – measured at 254 nm. Chromatogram B – measured at 325 nm.


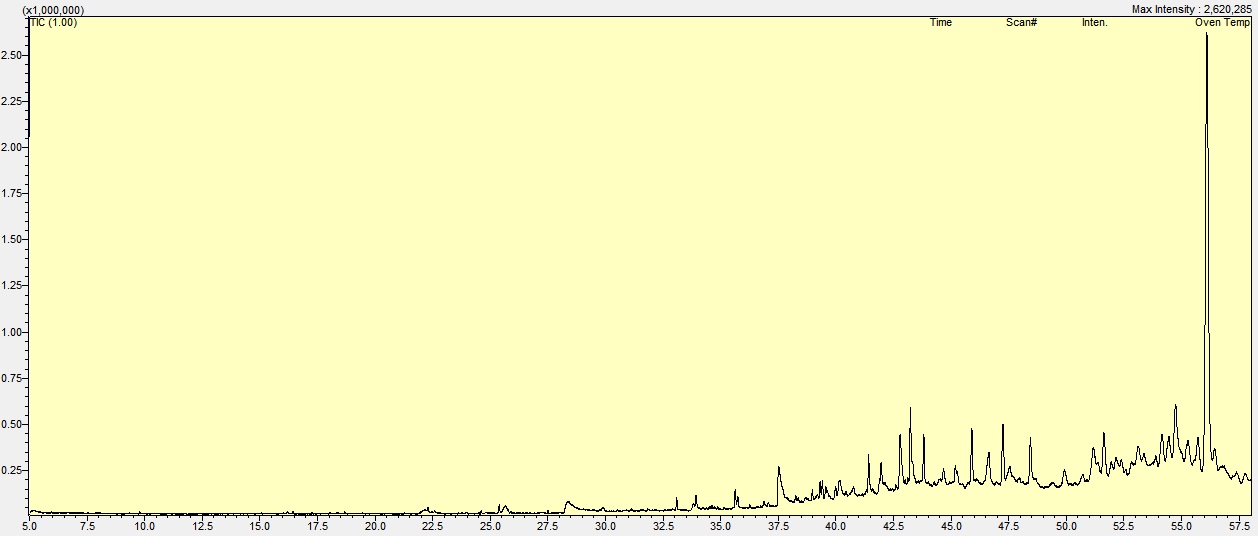


**Supplementary Figure S2.** TIC chromatogram of hydroethanolic extract of *S. sylvatica* L.


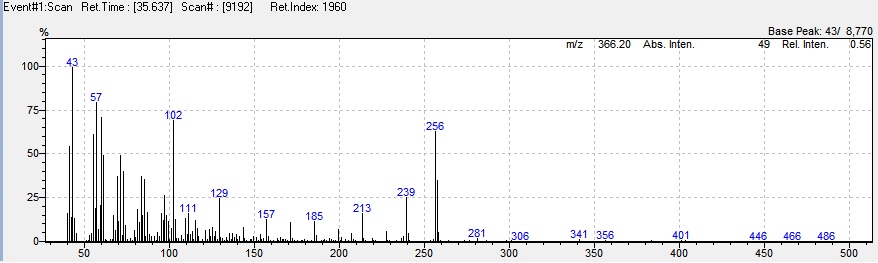
**Supplementary Figure S3.** Mass spectrum of unidentified diterpene (RI = 1960).


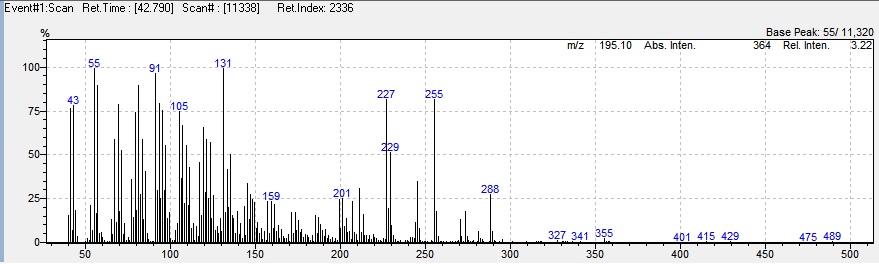
**Supplementary Figure S4.** Mass spectrum of unidentified diterpene (RI = 2336).

## Supplementary tables

**Supplementary Table S1**. MS/MS spectra of compounds identified with the use of HPLC-MS fingerprinting.

| *No* | *MS/MS fragments* | *Spectrum* | *Proposed compound* |
| --- | --- | --- | --- |
| 1 | 419, 315, 161 |  | Verbasoside |
| 2 | 191, 179 |  | Neochlorogenic acid |
| 4 | 595, 513, 463, 403, 295 |  | Kampferol triacetyl-pentoside-hexoside |
| 6 | 191, 173 |  | Chlorogenic acid |
| 8 | 191, 161 |  | Cryptochlorogenic acid |
| 10 | 509, 461, 299, 161 |  | verbascoside |
| 11 | 285, 191 |  | Luteolin glucoside |
| 12 | 327, 285 |  | Kaempferol hexoside |
| 13 | 509, 461, 299, 161 |  | Acteoside |
| 14 | 623, 463, 309 |  | Vebascoside derivative |
| 15 | 679, 623, 161 |  | Verbascoside derivative |
| 17 | 403, 357, 285, 187 |  | Luteolin derivative |
